# Physicians’ Mutual Benefit Association of Texas

**Published:** 1888-08

**Authors:** 


### Society Notes.

### PHYSICIANS' MUTUAL BENEFIT ASSOCIATION OF TEXAS.

OFFICERS.

President—Dr. W. A. Morris, Austin, Texas.
First Vice President—Dr. F. R. Martin, Kyle, Texas.
Second Vice President—Dr. M. A. Taylor, Austin, Texas.
Secretary and Treasurer—Dr. F. E. Daniel, Austin, Texas.

Board of Directors—Dr. R. M. Swearingen, Dr. J. W. McLaughlin, Austin; Dr. Geo. Cupples, San Antonio; Dr. D. C. Hewson, Orange; Dr. S. A. Stone, Richmond; Dr. W. P. Burts, Fort Worth; Dr. T. J. Bell, Tyler; Dr. R. H. Harrison, Sr., Columbus.

Executive Committee—President, Dr. W. A. Morris, Austin; Secretary and Treasurer, Dr. F. E. Daniel, Austin; Directors, Dr. R. M. Swearingen, Dr. J. W. McLaughlin, Austin; Dr. F. R. Martin, Kyle.

The Board of Directors have the general supervision of the business and the books of the Association, and order the levying of assessments and the payment of death benefits. The Executive Committee is to meet quarterly, and shall act in emergencies when it is not convenient to get a full meeting of directors.

The Board of Directors were instructed by resolution, at the meeting in Galveston, to issue a circular address to the profession. It will appear in the next issue.

Physicians are earnestly requested to become members at once. No formal application is necessary. Send in name of applicant and name of beneficiary, together with one dollar, which pays the initiation fee, or dues, for the current year, ending December 31st. On the first of January, each year, the sum of one dollar is again payable, the dues for that year, which sum goes to the expense fund, out of which all expenses are paid, including the Secretary's salary (as far as it will do so), stationery, postage, office rent, etc.; except the expense attending the collection of assessments. (This latter consists of the postal cards, and printing the same, and postage on return rceipts, exchange, or premium on money order, etc., and is deducted from the assessments, the net amount of collections going to the benefit fund, and is paid to the beneficiary for whose benefit the assessment is made, under order of the Board of Derectors.) There is *no other expense attending membership.* When a member dies, an assessment of *one dollar* is levied on each member for the beneficiary named by the deceased member. There have been only seven assessments (one dollar each) made during the four and a half years the Association has been in operation. And out of a small membership $684 has been paid to six widows, an average of $114 each, as will be seen from the following table:

| | |
|---|---|
| Certificate No. 26.—Paid Mrs. Park (61 members), net | $ 55 00 |
| Certificate No. 89.—Paid Mrs. Sayers (121 members), net . . . . . . . . . . . . . . . . . . . . | 109 00 |
| Certificate No. 134.—Paid Mrs. Welch (132 members), net . . . . . . . . . . . . . . . . . . . . | 120 00 |
| Certificate No. 46.—Paid Mrs. Kilpatrick (157 members), net . . . . . . . . . . . . . . . . . . | 143 70 |
| Certificate No. 25.—Paid Mrs. Starley (147 members), net . . . . . . . . . . . . . . . . . . . . | 135 50 |
| Certificate No. 105.—Paid Mrs. Glover (147 members), net* . . . . . . . . . . . . . . . . . | 121 00 |
| | $684 20 |
| Averrge of $114 each. | |
| Certificate No. 57.—Dr. Y. D. Harrington, assessment No. 7, now in process of collection, and will net, out of 147 members, about . . . . . . . . . . | $121 00 |
| | $805 20 |
| Or an average if $115. | |

One hundred and fifteen dollars is not much, but in the case of one of the beneficiaries above mentioned, it was a God-send. The widow, at a very advanced age, was left destitute, and dependent either on friends to take care of her in her old age, or, as she informed the Secretary, she "would be compelled to seek employment," and that, too, my brethren, with her husband's doctors bills in her pocket representing thousands of dollars, and days and nights and hours of her husband's valuable time, when, leaving her and home, he has answered every summons for miles around, knowing no fear nor fatigue, in the conscientious discharge of his professional duties—Christian duties, he regarded them. Think of it, my brethren. How much good your poor little one dollar can do, and you will not miss it. It will come your time—every ones time—sooner or later, and suppose

*Twenty-one forfeited.

—well, there is no use in talking; if the above facts possess not eloquence enough to convince. And yet, there are physicians whose autograph signature the Secretary has in his possession, to a solemn promise to pay one dollar to the widow of each deceased member when notified so to do, who have forfeited their membership by non-payment; who have ignored alike their pledge and their sacred duty, and the claim upon them thus incurred. Strange indeed.

We trust the profession will give a new impetus to this really charitable order, the nucleus of which one man has so strenuously endeavored to keep alive—and has kept alive—for five years, by his individual exertion.

